# 

**Published:** 1892-08-20

**Authors:** 


No. 308, Vol. XII.—August 20th, 1892.

[Registered at the General Post Office as a Newspaper for transmission in the United Kingdom and Abroad.]

Per Annum, 8s. 6d., post free.

Monthly Parts, 6d.; post free, 8d.

Ditto per Annum, 8s., post free.

### THE HOSPITAL

WITH EXTRA NURSING SUPPLEMENT.

No. 308. Vol. XII.] SATURDAY, AUGUST 20th, 1892 [TWOPENCE.

